# Editorial: Neuroprotection in Brain Hypoxia

**DOI:** 10.3389/fnins.2019.00212

**Published:** 2019-03-22

**Authors:** Matilde Otero-Losada, Francisco G. Wandosell, Luis Miguel Garcia-Segura, Francisco Capani

**Affiliations:** ^1^Instituto de Investigaciones Cardiológicas, Universidad de Buenos Aires, Consejo Nacional de Investigaciones Científicas y Técnicas, ININCA.UBA.CONICET, Buenos Aires, Argentina; ^2^Severo Ochoa Molecular Biology Center (CSIC-UAM), Madrid, Spain; ^3^Cajal Institute (CSIC), Madrid, Spain

**Keywords:** neuroprotection, brain hypoxia, pathophysioloy, pharmacology, genetics

Despite there is compelling preclinical evidence from laboratory models of brain hypoxia suggesting potential neuroprotective strategies, only scattered data are available from clinical studies. In this regard, a few candidate neuroprotectants have been little studied based on their antioxidant, antiapoptotic, anti-excitotoxic, immunomodulatory, and neurotrophic effects.

In parallel with clinical innovations, preclinical research initiatives are also identifying new animal models that more closely resemble the clinical course and pathology of neurodegenerative diseases.

Clarifying the specific mission of the brain cells involved in the damage/repair system in the examined animal models is important to define new therapeutic targets. Moreover, this is so considering that after hypoxic damage, detrimental events like excitotoxicity, pro-apoptosis, pro-inflammatory reaction, and others intermingle with the repairing events in both time- and cellular-dependent fashion. Identifying more reliable post hypoxia brain markers is mandatory to design and develop potential therapeutic interventions over the follow-up time after the hypoxic episode, improving translational research from the experimental observations to the clinical application.

Thus, a deeper understanding of the precise participation of neurons, glia, and endothelial cells by contrasting and comparing the outcome of studies using animal vs. cellular models might be expected to offer more clues for designing new therapeutic strategies to reduce the current gap among the experimental and the clinical data. One of the main issues should be to study the epigenetic mechanism of neuroprotective agents and their action on the genetic modification induced by hypoxia. Achieving this goal is presumed critical to obtain more conclusive results in patients that by now do not receive an appropriate therapy for mitigating the several diseases generated by brain hypoxia. Although a few ongoing studies are evaluating interesting approaches, future research is necessary to come to a novel mechanism of neuroprotection.

Here we summarize the contributing articles to our Topic conveying the goals and aims of the pertaining research.

- One of the chapters addresses the issue of metabolic syndrome (MetS) and neuroprotection (Etchegoyen et al.). An advanced outlook of MetS beyond its classical association with cardiovascular disease and Diabetes Mellitus Type 2 is revisited, for MetS also poses a risk factor for the nervous tissue and threatens neuronal function. The characteristically impaired metabolic pathways of MetS lead to hyperglycemia, insulin resistance, inflammation, and hypoxia, all closely associated with an overall pro-oxidative status. Oxidative stress is well-known to cause the wreckage of cellular structures and tissue architecture. Alteration of the redox homeostasis and oxidative stress alter the macromolecular array of DNA, lipids, and proteins, in turn disrupting the biochemical pathways necessary for normal cell function. The authors revise a few essential concepts of MetS pathophysiology, and explore some neuroprotective approaches in MetS concerning brain hypoxia. The prevalence of metabolic syndrome (MetS), a risk factor for neurological disorders, has drastically increased in developing countries over the years as a major byproduct of industrialization. Many factors, like high-calorie diets and a sedentary lifestyle, bolster the spread of this disorder. Undoubtedly, the massive still increasing incidence of MetS places this epidemic as an important public health issue.
- In the same line, another chapter discusses neuroprotection targeting protein misfolding on microvascular dysfunction and chronic cerebral hypoperfusion (CCH) in MetS (Herrera et al.). Persistent reduction in oxygen and energy supply to the brain implies brain hypoxia and consequent protein misfolding. This silent process represents a linking mechanism between CCH and Alzheimer's disease. Several experimental studies using distinct models of CCH revealed neurodegeneration was induced through protein misfolding. The regulation of proteostasis network pathways, such as ubiquitin-proteasome system (UPS), chaperone-mediated autophagy, and macroautophagy, appears as a novel target for neuroprotection. Lipoxin A4 methyl ester, Baclofen, URB597, N-stearoyl-L-tyrosine, and melatonin are potential neuroprotective agents to rebalance proteostasis network in CCH. Therapeutic drug options targeting misfolded proteins offer promising treatments for cognitive impairment following CCH in MetS.
- The neuroprotective role of palmitoylethanolamide (PEA) in perinatal asphyxia (PA) is also examined (Herrera et al.). The incidence of PA is estimated at 1/1,000 live births in developed countries and 5–10/1,000 live births in developing countries, causing not only mortality but also morbidity out of synaptic and cytoskeletal alterations associated with neuronal death. Cerebral palsy, epilepsy, and several neurodevelopmental disorders are common complications of PA. The role of PEA, known to protect in several models of brain injury and neurodegeneration, was studied in a well-established murine model of PA. Treatment with PEA successfully reversed neurodegenerative changes in hippocampus 1 month after experimental PA insult in rats. Data obtained using conventional electron microscopy (EM), immunocytochemistry, and immunohistochemistry for different markers of neuron and glial cells, ethanolic phosphotungstic acid (E-PTA) staining combining with electron tomography, and 3-D reconstruction techniques support the neuroprotective role of PEA in PA.
- Another chapter discusses the activation of the PI3K/Akt/GSK3 pathway by estradiol in long-term neurodegeneration following PA (Saraceno et al.). Based on their findings, the authors suggest that the interaction between ERα and the type 1 insulin-like growth factor receptor (IGF-IR) with the subsequent downstream activation underlies the beneficial effects of estradiol observed in late treatment of PA.
- The lack of impact of the selective inhibition of Janus kinase 3 (JAK3) on anatomical or neurobehavioral outcomes in experimental ischemic stroke is also addressed (DeMars et al.). The Janus kinase 3 (JAK3) is associated with the common gamma chain of several interleukin receptors essential to inflammatory signaling. The potential role of JAK3 in stroke-induced neuroinflammation was studied, and the effects of JAK3 inhibition with decernotinib (VX-509) on infarct size and behavior were investigated. Total and phosphorylated/activated JAK3 dramatically increased after stroke in the ipsilateral hemisphere in mice. However, JAK3 inhibition did not reduce infarct volume 48 h after stroke or altered behavioral outcomes sensitive to neurological deficits.
- Another chapter summarizes the state-of-the-art of the mechanistic target of rapamycin complex 1, mTORC1, in pathological situations, focusing mainly on ischemia/hypoxia (Perez-Alvarez et al.). Downstream the PI3K-Akt pathway, mTORC1 is deregulated after ischemia and neuronal oxygen-glucose deprivation (OGD). Neuroprotective intervention with estradiol or a specific AT2R agonist regulates mTORC1 activity, affecting VEGF levels.
- The antioxidative and antiapoptotic effects of delta-opioid peptide [D-Ala2, D-Leu5] enkephalin on spinal cord ischemia-reperfusion injury in rabbits (Fu et al.), the molecular bases of brain preconditioning (Deryagin et al.), and the effect of danhong (a complementary alternative medicine for chronic stable angina) combined with tissue-plasminogen activator in focal embolic stroke (Li et al.) are other captivating subjects discussed.

## Author Contributions

All authors listed have made a substantial, direct and intellectual contribution to the work, and approved it for publication.

### Conflict of Interest Statement

The authors declare that the research was conducted in the absence of any commercial or financial relationships that could be construed as a potential conflict of interest.

